# Self-assembled, disordered structural color from fruit wax bloom

**DOI:** 10.1126/sciadv.adk4219

**Published:** 2024-02-07

**Authors:** Rox Middleton, Sverre Aarseth Tunstad, Andre Knapp, Sandra Winters, Susan McCallum, Heather Whitney

**Affiliations:** ^1^ University of Bristol, Bristol, UK.; ^2^ Technische Universität Dresden, Dresden, Germany.; ^3^ Leibniz Polymer Institut, Dresden, Germany.; ^4^ University of Helsinki, Helsinki, Finland.; ^5^ James Hutton Institute, Dundee, UK.

## Abstract

Many visually guided frugivores have eyes highly adapted for blue sensitivity, which makes it perhaps surprising that blue pigmented fruits are not more common. However, some fruits are blue even though they do not contain blue pigments. We investigate dark pigmented fruits with wax blooms, like blueberries, plums, and juniper cones, and find that a structural color mechanism is responsible for their appearance. The chromatic blue-ultraviolet reflectance arises from the interaction of the randomly arranged nonspherical scatterers with light. We reproduce the structural color in the laboratory by recrystallizing wax bloom, allowing it to self-assemble to produce the blue appearance. We demonstrate that blue fruits and structurally colored fruits are not constrained to those with blue subcuticular structure or pigment. Further, convergent optical properties appear across a wide phylogenetic range despite diverse morphologies. Epicuticular waxes are elements of the future bioengineering toolbox as sustainable and biocompatible, self-assembling, self-cleaning, and self-repairing optical biomaterials.

## Teaser

The nanostructure of wax bloom of fruits like blueberries is responsible for their color and is reproduced in the laboratory.

## INTRODUCTION

Blueberries are observably blue; however, the pigments found in blueberries are not. Recent work demonstrates explicitly that the color variation of blueberries does not predominantly depend on pigmentation (*1*). The anthocyanins, contained in high concentrations in these fruits, generally have dark red scattering profiles. How then do fruits like blueberries and sloes produce their blue color? In many biological materials, nanostructures of nonabsorbing molecules interact with light via wave interference to reflect bright, highly saturated colors, creating “structural color” (*2*). Here, we analyze how the nanostructure of epicuticular wax, which arises by self-assembly (*3*), produces the coloration of a diverse group of “bloom fruits.” We find that the appearance is dominated by scattering from the random assembly of nonspherical particles and, unlike the coherent interference found in other structurally colored fruits that rely on periodic lengthscales within the material, is dominated by form factor contribution. We define bloom fruits as those with dark pigmentation and an epicuticular wax crystal coating, referred to as “bloom.”

**Fig. 1.**
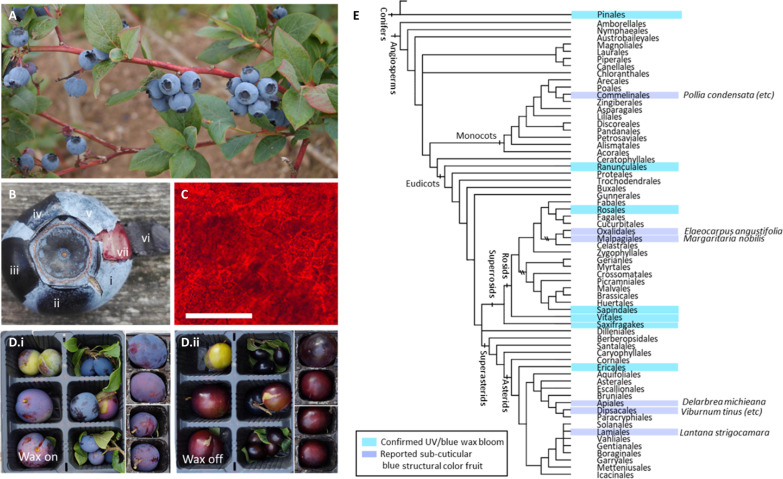
Structural color in wax bloom produces blue appearance on fruits across a wide phylogeny. (**A**) Undamaged highbush blueberries growing on the plant. (**B**) Blueberry with (i) unmodified wax, (ii) mechanical wax removal, (iii) chloroform wax removal, (iv) surface application of (almost) index-matching oil *n* = 1.518, (v) surface application of water, (vi) outer skin peeled to reveal flesh, and (vii) underside of peeled skin. (**C**) Transmission optical microscopy of peeled blueberry skin from internal edge showing red pigmentation in epidermal cells. Scale bar, 200 μm. (**D**) (i and ii) A selection of plum (*Prunus domestica*) fruits with different cell pigmentation, (i) with wax intact and (ii) with wax removed. (**E**) Confirmed occurrence of bloom disseminules across phylogeny. Previously known structurally colored (multilayer) fruits are shown in dark blue: *Delarbrea michieana* (*11*), *Elaeocarpus angustifolia* (*12*), *Pollia* species (*13*, *14*), *Margaritaria nobilis* (*15*), *Viburnum* species (*16*, *17*), and *Lantana strigocamara* (*18*).

Having a high discriminatory sensitivity to blue light is extremely common across vertebrates with color vision (*4*) and, in particular, visually guided frugivores, making blue discriminable or “chromatically salient” and therefore a valuable signaling color (*5*). However, identification of blue fruits is rare; in a broad global-scale study, blue fruits constituted a group so small that they were classed as outliers (*6*). This is in part explained by the relatively rare deployment of blue biological pigments (*7*). Blue-reflective pigments require absorption of lower energy photons by large, energetically expensive molecules (*8*). Blue pigment-derived color exists in plants, especially in flowers (*9*), and in some fruits such as *Dianella tasmanii* (*10*). However, these molecules are energetically costly, and structural color can offer a less costly route to blue color. A handful of other known blue fruits have been identified as structurally colored (see Fig. 1) (*11*–*18*). Blue-reflective fruits might be less rare than currently documented, as bloom fruits have been overlooked in color surveys, perhaps because their strong anthocyanin pigmentation develops with ripeness (*19*) but, reflecting red/black, does not determine their blue ultraviolet (UV) color appearance (*1*, *20*).

Plant epicuticular waxes are self-ordering and self-repairing (*21*) surface coatings that can determine properties of the plant surface such as self-cleaning, wettability (*22*), and resistance to insect and microbial pathogens. Plant waxes share with biomaterials like cellulose a very wide and multifaceted range in biological function (*21*). An extensive body of work on epicuticular wax crystals exists. It is largely focused on hydrophobicity properties but has identified epicuticular wax crystals as developing external to the plant cuticle through self-assembly from crystallization (*23*, *24*), which can be reproduced in many (but so far not all) instances in the laboratory (*3*), including complex coiled structures.

The optics of epicuticular waxes has been the focus of surprisingly little analysis. An exception is a demonstration of thin-film interference producing a gold sheen on *Tradescantia* leaves (*25*). High UV reflectance in *Picea pungens* was reported in 1975 (*26*) along with the reflectance spectrum and later the UV reflectance from other leaves (*27*). Although the *P. pungens* mechanism was not discussed by the authors of that paper, it has since been generally attributed to Rayleigh scattering (*28*). Rayleigh, Tyndall, and Mie scatterings refer to single-particle scattering effects, with different degrees of precision (*29*). While all nonmolecular, non-iridescent color effects were once generally attributed to this mechanism (*30*, *31*), the imprecision of its application in dense biomaterials [in comparison to dilute suspensions and atmospheres, where it is well understood (*32*)] has meant that many of these assumptions have been overturned (*33*). Despite the general assumption, the color of bloom fruits was not previously listed prominently in biomaterials attributed to Rayleigh scattering.

Mie scattering, the analytical result for single scattering from spheres and infinite cylinders 4(*34*), has attracted particular interest, in part because of its analytic solution but also because of its application in producing vibrant spherical-colloid colorant materials (*35*). Mie resonance in spherical particles produces coloration corresponding to particle diameter, allowing peaks across the optical spectrum. Theoretical analysis of Mie scattering has investigated the transition between single-particle scattering and assemblies with resonance or multiple scattering, as dense thick assemblies transition to sparse thin ones (*36*).

Study of Mie assemblies as disordered photonic glasses have shown that the optical response can be broken down into a “form factor” contribution from individual particles, and a “structure factor” contribution from their spatial relationships to one another. Although negligible in photonic crystals, a form factor contribution appears in photonic glasses due to departure from strictly periodic scattering centers. Analysis of this has shown that form factor, as a minority contribution to overall reflectance, limits purity of non-blue colors (*37*). It is known that although multiple scattering is important, in the low-scattering limit of very thin samples, the spectral response from individual particles can qualitatively predict spectral shape (*38*). Analyzing the interaction of light with epicuticular wax expands our understanding of the materials, advancing their potential for application. Biomaterials have huge value in sustainable and biologically compatible bioinspired engineering applications. For example, research into the optical and self-assembly properties of cellulose has led to an explosion in innovation in coatings, sensors, and colorants (*39*). Epicuticular waxes show particular promise due to the wide range of morphologies (*40*) the self-assembled crystals take, as well as the range of biological adaptation in environment and function.

## RESULTS

### Epicuticular waxes produce structural color across a wide phylogenetic range

Highbush blueberry (*Vaccinium corymbosum)* fruit is used in Fig. 1 (A to C) to demonstrate the epicuticular wax bloom characteristics. The fruit appears blue in trichromat human vision (Fig. 1, A and Bi), but the color is removed by mechanical abrasion of the surface (Fig. 1Bii), rinsing with chloroform (Fig. 1Biii), and application of immersion oil (*n* = 1.5) (Fig. 1Biv), while wetting with water leaves it unaffected (Fig. 1Bv). The pigment in the epidermal cells is very dark (Fig. 1Bvi) but, when released by cell breakage as juice, appears red over the whitish flesh (Fig. 1Bvii). Transmission optical microscopy under very high intensity light confirms the red color (Fig. 1C) of the epidermal cell pigment, although its strong absorption makes the macroscopic appearance of ripe dewaxed blueberry surface black. The dissolved epicuticular wax is transparent in (chloroform) solution over the visible range. Spectrophotometry shows a strong absorption in UV below 300 nm for blueberry wax, absent in other species (fig. S1), which accounts for a sub–300 nm reflectance drop-off observed only in blueberries. Color arising from structure is nonabsorptive and, therefore, semitransparent. Wax bloom, in common with other structural color, therefore requires an underlying absorptive background to be the sole source of coloration. Reflectance from dewaxed surfaces and exposed pigmented skin is shown in fig. S2. It also produces mixed colors in conjunction with non-black pigmentation, as seen in a range of plum cultivars in Fig. 1D. In many such cases, wax bloom still contributes substantially to the overall color. This is important both in fruits, in which strong color mixing occurs, and in the transition of appearance of unripe fruits, which generally develop from mixed green-UV through red-UV to blue-UV during maturation (fig. S3). We identified these traits of bloom fruits in fruits (and in conifers, cones) from species across seven phylogenetic orders, which are indicated in Fig. 1E.

**Fig. 2.**
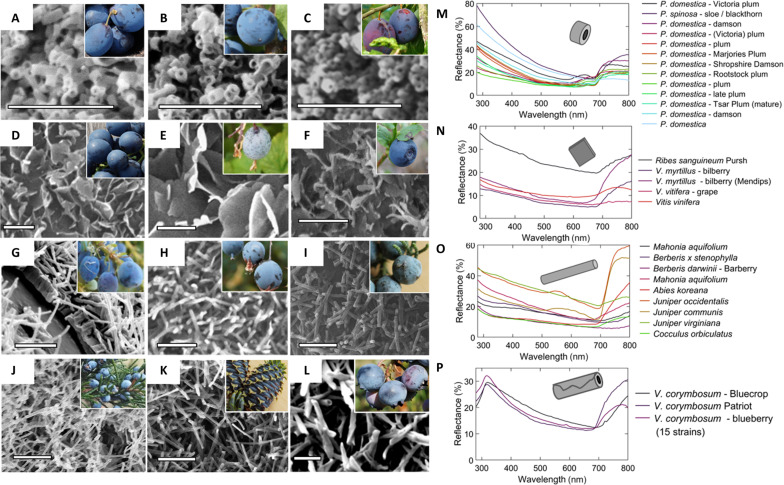
Wax morphologies differ strongly between species, but spectral signatures do not. (**A** to **L**) SEM images of wax bloom crystals; scale bar, 2 μm. Photo insets: (A) Damson *P. domestica*. (B) Sloe, *Prunus spinosa*. (C) Victoria plum *P. domestica*, ssp. *Intermedia*. (D) Grape *Vitis vinifera* “Hamburg.” (E) Flowering currant, *Ribes sanguineum* Pursh. (F) Bilberry *Vaccinium myrtillus*. (G) *M. aquifolium*. (H) Barberry, *Berberis darwinii*. (I) *Juniperus occidentalis*. (J) *Juniperus virginiana*. (K) *Abies koreana*. (L) Blueberry *V. corymbosum* “Rocio.” (**M** to **P**) Average spectra from each species by morphology group. (M) Ring spectra. (N) Slab spectra. (O) Rod spectra. (P) Tube/blueberry spectra.

For each species, we examined the morphology of the epicuticular wax crystals by scanning electron microscopy (SEM), for which a range of species are shown in Fig. 2. Epicuticular wax crystal morphologies vary widely between genera according to chemical composition and are commonly categorized by shape (*41*, *42*). Here, we used four shape categories (“ring,” “rod,” “slab,” and “tube”) to capture the characteristic dimensions plotted in fig. S4. Figure 2 (M to P) shows surface optical reflectance spectra grouped by wax morphology. Despite the major differences in the particle shape, the spectra share a monotonic inverse relationship between reflectance intensity and wavelength. The same or similar optical spectra are produced by waxes from diverse families of wax morphologies. The phenomenon shows apparent convergence in spectral reflectance across the phylogenetic range, despite morphological variation in the wax particles.

### Epicuticular wax spectra are dominated by random scattering from particles rather than periodic or quasi-periodic structure.

**Fig. 3.**
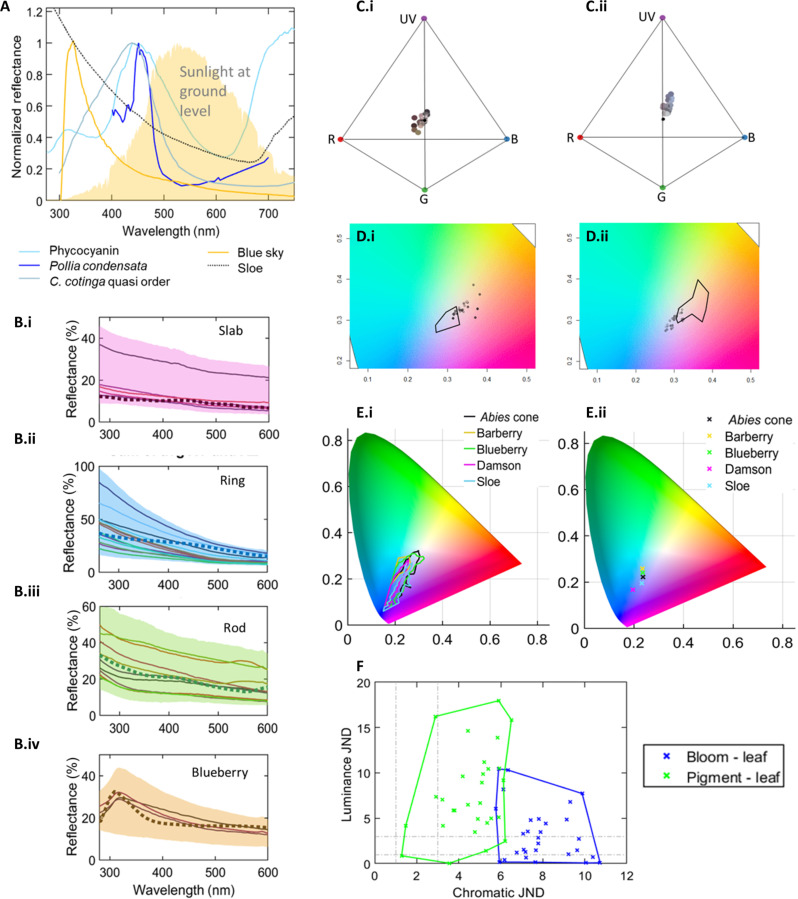
Epicuticular wax shows no visual-wavelength peak but is visually salient in both blue and UV channels for avian frugivores, indicating potential value in chromatic contrast. (**A**) Normalized reflectances compare spectral shape. Structural color [highly ordered Pollia, quasi-ordered *Cotinga cotinga* (*28*)] and pigment (phycocyanin) compared to sloe bloom and irradiance measured from blue sky. Sunlight at ground level explains the blue-sky cutoff below 300 nm. (**B**) (i to iv) All spectra (solid lines) and 90% confidence intervals (shaded areas) are shown along with the single-particle scattering model (thicker dotted/dashed lines) from FDTD scaled to the peak intensity measured. In (iv), the blueberry model also includes the absorption below 300 nm measured in blueberry wax, as shown in fig. S1. The scattering profiles from FDTD are scaled to match the intensity of the measured spectra, by slab: 3 × 10^14^; ring: 5 × 10^14^; rod: 3 × 10^15^; blueberry: 3 × 10^14^. Further variation of model parameters is in fig. S6. (**C**) (i and ii) Projection of the bloom fruits with (i) wax intact and (ii) removed, into tetrachromat *Turdus merula* (Blackbird) visual space. (**D**) (i and ii) Projection of the bloom fruits with (i) wax intact and (ii) removed, into trichromat human CIE color space. Outlines show the range in the other plot. (**E**) (i and ii) Color from calibrated photos of five fruit surfaces are plotted in CIE space. (i) Bound of points from the photographed fruit body. (ii) Each fruit’s average. The range contains the palest blue reflectance point, plotted from specular reflectance in (Di), and also shows the resultant full visible color range. (**F**) Chromatic and achromatic just noticeable differences (JNDs) (*62*) show the discriminability of fruits with wax intact (bloom-leaf, shown in blue) and wax removed (pigment-leaf, shown in green) against green foliage. One JND (discriminable in bright lighting) and three JND (discriminable across lighting conditions) thresholds are indicated with gray dashed lines.

The comparison of bloom reflectance spectra with other spectral signatures, as in Fig. 3A, shows a clear distinction between bloom and other characteristic reflectance spectra. Other kinds of biological pigmentary color, and structural color (both ordered and disordered), show a peak in the visible wavelength range (corresponding in general to the dominant visual appearance). Contrastingly, bloom spectra, in common with the spectrum of the blue sky (produced by Rayleigh scattering), have a monotonic inverse relationship between wavelength and reflectance (*29*).

Spectral peaks from structural color arise from constructive interference of light waves with nanostructure arrays that have a characteristic lengthscale corresponding to the reflected peak wavelength. In photonic crystals, this characteristic lengthscale occurs on crystal planes and produces angle-dependent coloration (*2*). In photonic glasses, directional disorder occurs between scattering loci, but a characteristic inter-locus distance is preserved, and thus, constructive interference and a reflectance peak is also preserved but without angle dependence (*43*). The analysis of disordered photonic glasses (*37*) has shown that the disorder introduces additional scattering, designated form factor. This depends on individual scatterers, rather than their relationship to each other, which constitutes the structure factor. Form factor corresponds to the scattering of light by individual nonspherical particles of sub- or near-wavelength size. In dilute systems, single-particle scattering has been identified since the late 19th century as “Rayleigh” scattering or as a “Tyndall” phenomenon in which a monotonic relationship between wavelength and scattering is observed, where shorter wavelengths are scattered more (*29*). As the analog in dense biomaterials, single-particle scattering as form factor contributions in photonic glasses also has this bias, with high relative reflectance in blue wavelengths therefore limiting the non-mixed color palette of photonic glasses to blue (*37*). Other colors of photonic glasses may be produced, but where disorder makes it a “glass” rather than a crystal, the color is usually mixed, appearing light or nonsaturated rather than saturated (*44*).

The lack of a reflectance peak in bloom reflectance spectra is notable because it indicates a lack of constructive interference in the material, in turn a sign of no characteristic lengthscale order (*2*). We made Fourier transforms and structure measurements from the EM measurements of bloom wax in cross section and normal to the surface. These indeed showed no sign of short- or long-range order. Wax crystals have different uniform stereotypical shapes, distributions, sizes, and thicknesses depending on species (fig. S4), but none show evidence of characteristic spacing between adjacent particles.

To understand the case in which coherent interference could nevertheless explain the result (i.e., to construct a plausible structure factor model depending on packing of the regular particle dimensions), we constructed a one-dimensional (1D) model of multiple scattering from very tight, disordered packing of ring particles. In this model, a peak in the reflectance spectrum could occur at sufficiently low wavelength that is not measured, and the measured spectral tail is interpreted as a monotonic spectrum. With assumption of very close packing and actual dimensions smaller than measured (i.e., assuming strong presence of artifacts in measurement), the closest fit model shows a loose approximation to the observed spectra for ring particles (fig. S5), although it has a flattened profile at low wavelengths, which is not observed. Furthermore, such packing would only be feasible with some (high symmetry) crystal shapes, despite experimentally similar measured optical responses between different bloom wax crystal morphologies. We used the same approach to confirm that the conformation of thin-film fringes predicted by the parameters measured do not provide adequate explanation of the effect (also fig. S5).

Since we could identify no contribution from a periodic structure factor of the highly disordered nanostructure, we modeled the spectra as arising from form factor contributions, as modeled in previous low-scattering approximation (*38*). We used a Monte Carlo approach, averaging over many single-particle scattering events, modeled using computational Maxwell equation solver [finite-difference time-domain (FDTD)]. This approximation requires the assumption of small scattering cross section and thin layer thickness so that the impact of scattering events on the EM field is negligible with respect to the incident wave.

For the low-multiple scattering regime, the structure thickness must be smaller than or similar to the transport length or distance traveled by light through the material without scattering. This assumption is suggested by previous work on a similar system in which multiple-scattering models break down for thin films with thickness less than the transport length of the material (*36*). In the work referenced, a dense Mie particle assembly of polystyrene spheres between 94 and 138 nm in diameter is analyzed. The transport length can be calculated analytically for spherical particles and was found in that work to be ~8 μm. Reflectance from a film thickness of 6 μm was found to depart from multiple-scattering assumptions.

Taking this transport length as a starting point, polystyrene has a refractive index of around 1.6 (slightly larger than the refractive index for wax assumed here) (*45*). The epicuticular wax shapes also have a far reduced geometric cross section with respect to a filled sphere. Both these features serve to reduce effective volume of interaction between particle and light, increasing the transport length (*34*). Last, the thickness of the material present on bloom surfaces is found to be 2 to 4 μm, larger only in sparse rod structures. We therefore expect the departure from a strictly multiple-scattering model to be appropriate in epicuticular wax nanostructure, until a much greater thickness is observed. Instead, as in (*38*), we used a scattering model in this instance, which analyzed individual particles (whereas in the previous work this was spheres, our particles are nonspherical). The models are shown with the experimental data in Fig. 3B (i to iv). Despite its limitations, the single-particle model approximates the measured spectra qualitatively and supports the dominant role for form factor contribution in the scattering profile, analogously to the successful model of spectral shape through spherical form factor models (*38*) and the blue-bias minor contribution identified in photonic glass arising from form factor (*37*). Although there is no spectral peak in the visual wavelength range, the activation of visual channels in UV and in blue, and its differential with longer wavelength receptors, is sufficient to produce chromatic salience both in a tetrachromatic avian (blackbird) visual model (Fig. 3C, i and ii) and in human trichromatic vision, plotted in CIE space (Fig. 3D, i and ii). The projection of the specular reflectance shows that the fruits are all blue when waxy and dark/red when dewaxed. The color appears desaturated when projected from spectral profile. For dewaxed fruits, this is because they were very dark (low reflectance in all wavelengths); however, the relatively low saturation for waxed fruits is due to the lack of a peak in the spectrum. Nevertheless, under natural lighting conditions, the fruits appear to human trichromat vision relatively saturated. Color spaces appearing across fruit surfaces extracted from color-calibrated photos and plotted into CIE space in Fig. 3E (i and ii) show that the visual appearance actually encompasses a range across the unevenly lit surface, with both more tones and the unsaturated tones (in Fig. 3C, i and ii) measured by specular optical-axis reflectance.

Fruit coloration visibility is strongly affected by background color. The contrast of the fruits with respect to green foliage in both luminance and chromaticity (for the specular spectra) is therefore plotted in Fig. 3F, comparing fruits with wax bloom and after wax is removed to reveal underlying pigment. Although waxy fruits have lower luminance discriminability (the luminance of a waxy fruit is closer to a leaf than a dewaxed one), the chromatic discriminability is strongly enhanced (the color contrast of bloom fruits with green leaves is highly discriminable and much higher than dewaxed fruits).

### Replication of structural color from fruit bloom

**Fig. 4.**
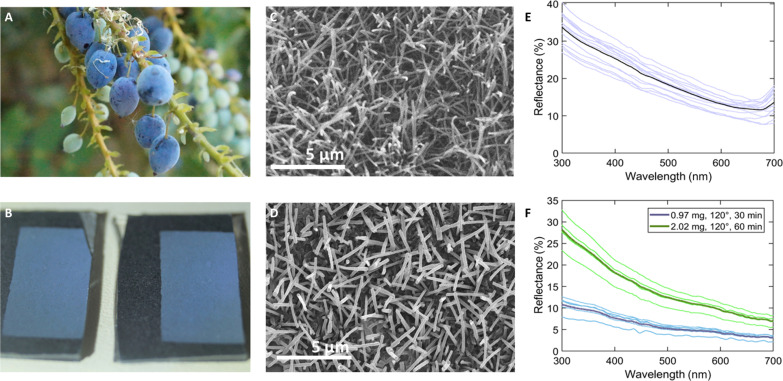
Reproduction of structurally colored wax by in vitro recrystallization. (**A** and **B**) Photographs of (A) *M. aquifolium* fruits and (B) redeposited *M. aquifolium* wax (left 1.75 mg, right 2.02 mg, both deposited over 1 hour at 120°C). (**C** and **D**) SEM images showing the crystal structure of epicuticular wax in (C) fruit surface and (D) redeposited sample. (**E** and **F**) Spectral measurements from (E) fruit surface and (F) redeposited samples; legends show initial wax weight, crucible temperature, and deposition duration.

We extracted epicuticular wax from *Mahonia aquifolium* fruits (Fig. 4A) by fast dipping in chloroform to extract only the outer (epicuticular) layer of wax (*46*). We recrystallized the wax via thermal evaporation and deposition (*24*) under vacuum onto non-chromatic black card (Fig. 4B). The wax, having been transparent in solution (fig. S1A) and white as a solute remaining after solvent evaporation, self-assembled after deposition to replicate a similar nanostructure (Fig. 4, C and D) and corresponding blue coloration (Fig. 4F) close to the spectral measurement from naturally occurring *M. aquifolium* fruit surfaces (Fig. 4E). The production of blue color by nanostructure is therefore demonstrated. Increased deposition time and weight showed a corresponding increase in the reflectance brightness of the deposited coating.

## DISCUSSION

This work adds the widespread matte blue–UV chromaticity of bloom fruit to the known photonic effects that occur naturally in epicuticular wax. This includes the gold sheen on *Tradescantia pallida* leaves (*25*), whitish “glaucous” wax on succulent leaves (*47*), and highly UV-reflective coating on *P. pungens* needles (*26*)*.* The known range shows already that wax nanostructure has a versatile range of photonic effects. The reproduction of the effect here [through already established techniques (*3*)] is a promising indication that other wax optics might also be accessible through recrystallization, and through adapting morphology and orientation of the structures, as in other photonic biomaterials. The prominence of the effect reported here indicates that there may be much more to understand about the range of optical phenomena in wax nanostructures. Further, the analysis indicating the importance of form factor in the effect produces a lens on both the discriminate fine-tuning of structures that has produced the convergent coloration in these instances and notable flexibility in morphological groups in which it has arisen.

The identification of visually salient blue-UV structural color from wax bloom demonstrates structural color in a much wider range of fruits than just those with subcuticular structure. The phylogenetically widespread occurrence indicates that it has evolved multiple times, likely many more than reported here. Its identification in part resolves the paradoxical situation of very few fruits seemingly using effective blue signaling colors, as structurally colored bloom overlaying dark anthocyanin pigmentation to produce blue-UV chromatic reflectance is widespread. The spectral similarity indicates potential convergence, despite diverse structures. This mirrors the convergence of appearance observed in petal striations to produce a blue sheen in flowers (*48*). We see therefore a second example of structural color in plants showing broad taxonomic stability, despite disparate structures. In both cases, a high degree of disorder plays a key role in the color produced and robustness to differences in that structure within a bounded range. The clear relevance in both cases to plant-animal interaction through visually guided foraging is also notable.

The use of structural color here overcomes the challenge that the dense, nutritionally valuable anthocyanins make fruits dark and therefore potentially inconspicuous (*49*). As observed also in *Viburnum tinus* (*16*), once overlaid with bloom, darker anthocyanins enhance the chromaticity of the fruit by reducing color mixing. This is also important in maturation (fig. S3): Bloom is present throughout fruit development, displaying a bright UV signal, but only once the bright pigmentary scattering of early ripening stages is removed is the final blue-UV mixed color, due entirely to the bloom structure, visible. Given the other functional benefits of bloom for plant health (*40*), there is also the possibility that its chromatic visibility could act as an honest signal, displaying an innate high-quality trait. The phenomenon is observed in unrelated cultivated and wild fruits, indicating convergent evolution in a signaling organ, but given the complexity of fruit ecology, behavioral tests would be required to understand whether the coloration enhances frugivore attraction.

Behavioral tests of the impact of bloom on bird attraction have reported reflectance spectra of some species included here, although the aim was not to understand its origin. Moreover, the studies focused specifically on UV reflectance. From this work, it is clear that the UV reflectance is visually salient to some birds, but in short-range “cafeteria experiments,” it is not clear whether UV itself enhances attraction to birds (*50*, *51*). The sloped monotonic spectral signature allows the coloration to be salient in both UV and blue visual channels. Crucially, it also features lower stimulation of longer-wavelength visual receptors, making it “colored” for both tetrachromats and trichromats, rather than white. Although these colors could in some cases be added by bright underlying pigments, in bloom fruits, the underlying pigment is dark and absorptive.

The proposed mechanism extends the role of form factor contribution in structural color, from prominent as described in photonic glasses (*37*), to primary in its role in these (2 to 6 μm) coatings (fig. S7). Although thin, this thickness is of the same order as structural color with coherent interference like multilayers (*2*), which justifies the analysis of its structure factor. This mechanism is analogous to resonant Mie scattering in spherical particles (*52*), the fabrication of which is of contemporary interest but shows the prominent role of scattering from nonspherical particles in dense materials.

The very simple optical model suggested here is successful, and we propose that, analogously to dominance in disordered photonic glass of single scattering from Mie spheres, it produces insight into color production in these ultrathin materials. However, there is much more to be understood about the materials and the models, which should form the basis of future work, and a full theoretical investigation into the range of architectures observed to produce the effect. The most important question is in the role of multiple scattering. There must be a role for multiple scattering in these materials, as the density of the scatterers implies interaction between them. Moreover, as nicely demonstrated in the Ewald sphere construction (*53*), transport length decreases with decreasing wavelength, making multiple scattering increasingly likely. The role of “imperfect shapes” and highly disordered nearest-neighbor relationships we suggest are particularly of interest. Given the spectral profiles identified and previous work on multiple scattering (*54*), we would expect contribution from multiple scattering also to be entirely disordered. Unlike in a photonic glass, where the disorder has a visible-lengthscale average coherence length, because of the highly irregular shapes, we expect the disorder in this case to produce greater scattering with shorter wavelengths far into the UV. Therefore, wax bloom is an apparently unusual example in biological coloration beyond whiteness, where truly random particulate scattering dominates, although its pervasiveness indicates that there may be other unexamined examples of the effect in biological materials. The lack of periodic structure factor contribution could suggest enhanced disorder of the material, as in *Cyphochilus* beetles*,* where the disorder is hypothesized to be enhanced to ensure that no coherent interference effects are present (*54*).

The simple reproduction of such self-assembled nonspherical particles in wax crystals is a direct benefit of working with the biomaterial itself, and a potential that could also be extended to other nanoparticle fabrication. Recrystallization of epicuticular waxes through thermal deposition in vacuum is a well-established technique that has been used to create complex hierarchical architecture (*24*). Although vacuum thermal evaporation does not replicate natural biological crystallization conditions, it allows wax molecules to reassemble without obstruction by additional forces. Understanding how this can be achieved outside of vacuum conditions for different waxes, as occurs on biological surfaces, should be the subject of further work.

We present here an instance of structural color from wax, which indicates the potential for a wider range of photonic effects arising from epicuticular wax (for example, in albedo-enhancing white coatings). Recrystallization of the structurally colored bloom directly demonstrates the bio-replicative engineering application. Both disordered photonic glasses (*55*) and single-particle spherical Mie scattering materials (*44*) have been produced in artificial materials, and the scalable production of these is the focus of ongoing research (*56*). Biologically derived structural color offers a sustainable, biocompatible, multifunctional approach to self-assembled, self-healing, and self-cleaning colorants and coatings. Epicuticular wax, in particular, has multiple known functionalities (*40*), making its applications potentially tunable to different optical/industrial applications. The straightforward self-assembly (*57*) and range and tunability of wax nanostructure (*42*), which have allowed for the coloration both observed and reproduced here, indicate a rich potential for application in nonabsorptive photonic surfaces.

## MATERIALS AND METHODS

### Materials

We identified and collected samples from botanic (Edinburgh, Dresden) gardens and private and community gardens in Bristol (UK), public access land, and the James Hutton Institute polytunnels or commercially. Shop-bought samples were compared to samples grown in known conditions, and no artifacts from commercial surface treatments were observed. We removed wax from the surface of the fruits using either gentle mechanical abrasion with paper tissue or wiping with tissue soaked in chloroform. Mechanical rubbing produced a smooth, specular reflecting surface, while chloroform dissolution produced a matte surface. This difference is because mechanical rubbing removes the relevant particulate epicuticular wax while leaving intact underlying smooth wax that is fused with intracuticular wax that permeates the cuticle. The surface produced is a polished artifact of rubbing. Using chloroform strips the relevant epicuticular wax back and may remove part of the outer layer of intracuticular wax as well. This is an important concern and is fully explored in previous literature (*46*). This, too, produces an “artificially textured” surface but not a smooth polished one as with rubbing. This affected the dewaxed spectra as shown in fig. S2.

### Imaging

We took macroscale photographs using a Sony a300 DSLR and Sony SAL30M28 macrolens and x-rite ColorChecker for fruit image calibration (processed using MATLAB Image processing toolbox). For spectral measurement, we used a DH-2000-BAL OceanOptics lamp, QR400-7-UV-Vis double-ended fiber, and an Avantes Flame UV-Vis Spectrometer. We calibrated it on an Avantes white diffuser standard. We took reflectance measurements using a metal mount-block holding the fiber normal to the surface (tangent to the fruit surface in the case of round fruits) at 7-mm distance. Spectra shown in Fig. 2 are averages across samples, smoothed over λ = 7.5 nm. Immersion oil was Zeiss Immersol 518. We prepared samples for SEM by mounting them on carbon tape and coating them with 5-nm gold in a Quorum Q150RES sputter coater; then, we imaged them in Zeiss Evo 15 ESEM under vacuum (standard settings: 10 kV, 50 pA).

### Visual modeling

For visual modeling, we used pavo version 2 (*58*) in R version 4.1.2. We used visual models (*4*) to convert spectral reflectance measurements into quantal catches to analyze colors as perceived by trichromatic humans and tetrachromatic avian seed dispersers (represented by the European blackbird *Turdus merula*). We implemented a D65 illuminant to model daylight conditions in the Von Kries model. We averaged spectral reflectance measurements for each species and smoothed them using a span of 8 nm. We modeled Blackbird vision (using vismodel) based on five photoreceptors [peak sensitivities: 557, 504, 454, 373, and 557 nm; cone cutoff wavelengths: 570, 515, 414, 330, and 439 nm; oil droplet types: R, Y, C, T, and P; cone types: long (LW), medium (MW), short (SW), and UV cones, which perceive color, and double cones, which perceive luminance] (*59*, *60*).

To determine the detectability of fruit against the background, we calculated just noticeable differences (JNDs). One JND is the discrimination threshold between similar colors in ideal conditions, calculated using the receptor noise limited model (here, JNDs are calculated pairwise between each fruit with wax both on and off and an average blueberry leaf spectrum (a characteristic chlorophyll-dominated green leaf), using relevant cone ratios; blackbird: 1:1.78:2.21:1.96 (UV:S:M:L) and Weber fraction [blackbird: *w* = 0.1 (*58*)]. The averaged blueberry leaf spectrum is used as an approximate model of background foliage for all fruits.

### Optical modeling

We made optical models using Lumerical 2019b Finite Difference IDE, a commercial Maxwell equation solver. We estimated the refractive index of the particles as 1.47 (*61*). This is a low-precision assumption, and future work should aim to improve precision on the dispersive refractive index. Absorption of several waxes was measured (see fig. S1A) and found to be extremely low, except for blueberry waxes below 350 nm. They were therefore treated as negligible and complex refractive indices were not used in the FDTD model, although in the blueberry model they were applied afterward, as shown in fig. S1B. We performed simulations with a source that averaged over two orthogonally polarized pulsed plane wave sources (160 to 600 nm) and the far-field scattering integrated over the 180° backscatter direction. No planes of symmetry were introduced, and simulation area boundary conditions were perfectly matched layer (PML) absorbing boundaries. We used individual 3D particles oriented within the simulation volume with varied orientation of the target particle with respect to the k-vector according to orientations observed in structural analysis and then averaged the full backscattered spectra.

### Wax extraction and redeposition

We extracted plant wax dominated by the fraction of epicuticular wax from fruits by fast (~2 s) dipping in chloroform (*46*). We filtered the solution (Fisherbrand grade 100 filter paper) and evaporated it to leave a dry white residue under a gentle stream of nitrogen. We loaded the 2 mg of the wax into an aluminum crucible in an adapted Balzers MED-010 thermal evaporator and decreased the pressure to 5 × 10^−5^ mbar. The crucible was heated over 2 min and held at 120°C for 30 min or 1 hour under vacuum. The 1 cm–by–2 cm flat substrate was held face-down 2.5 cm above the crucible. After evaporation, we released the chamber quickly (over approximately 10 s) to room temperature and pressure. On removal from the device, the coating is immediately visible, indicating either crystallization in the device under vacuum or relatively quickly during the fast repressurizing of the device on opening.

## Supplementary Materials

### This PDF file includes:

Figs. S1 to S7 References
