# Supplementary material for: Self-assembled, disordered structural color from fruit wax bloom
Source: Sci Adv. 2024 Feb 7;10(6):eadk4219. doi: 10.1126/sciadv.adk4219 (PMC10849586; doi:10.1126/sciadv.adk4219)
Supplement: Supplementary file 1 — Figs. S1 to S7 References [file sciadv.adk4219_sm.pdf]

Science Advances
AAAS

Supplementary Materials for

Self-assembled, disordered structural color from fruit wax bloom

Rox Middleton *et al.*

Corresponding author: Heather Whitney, heather.whitney@bristol.ac.uk; Rox Middleton, r.middleton@bristol.ac.uk

Sci. Adv. **10**, eadk4219 (2024)
DOI: 10.1126/sciadv.adk4219

This PDF file includes:

Figs. S1 to S7
References

Supplementary Material

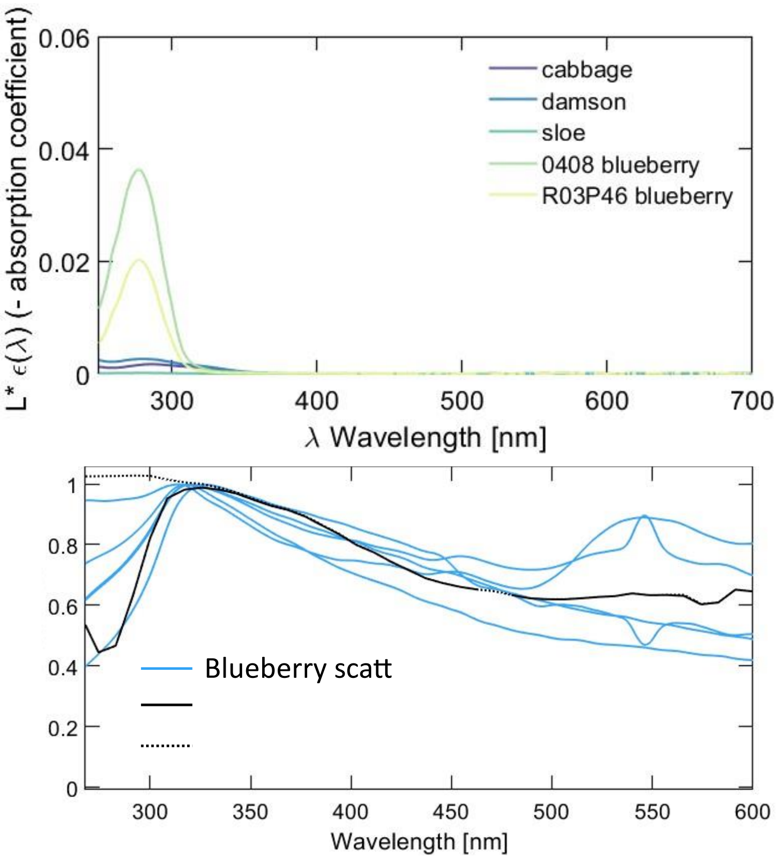

Fig. S1. The effect of absorption on blueberry reflectance spectra. (**A**) Absorption coefficient (multiplied by spectrophotometer transmission distance 1 cm, constant for each sample) of five different wax samples. Measured by spectrophotometry of waxes dissolved in $CHCl_3$ in a concentration series. Spectrophotometry demonstrates no visual pigmentary absorption although in blueberry wax, a strong sub-300 nm UV absorption is observed (which is negligible in other plant waxes). This is likely due to triterpenoid double bonds(63). **(B)** Blueberry wax absorption was applied to the tube model single scattering optical model in Fig.3.

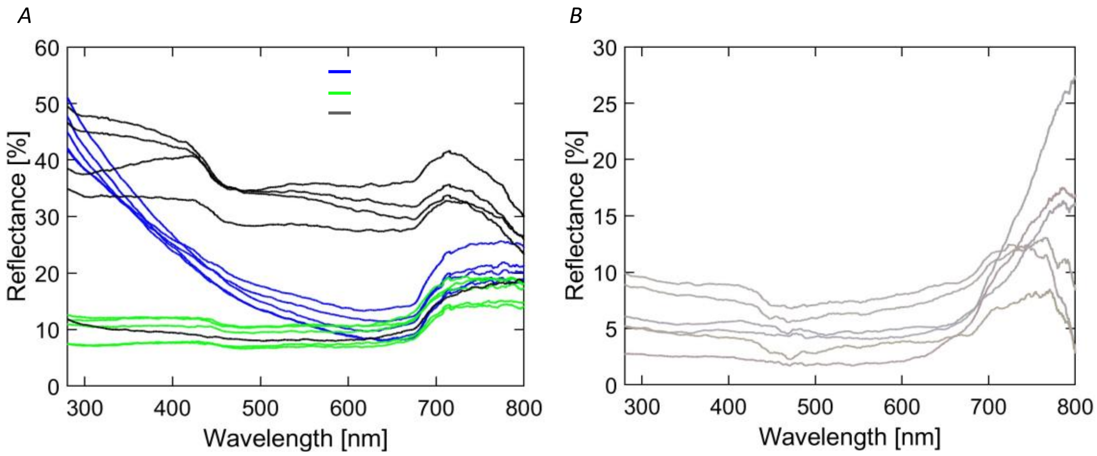

Fig. S2. Optical measurements of pigmented surfaces of damson and barberry a) Spectra from damson with wax intact and removed, comparing two wax removal techniques. Fast dipping in chloroform ($CHCl_3$), and mechanical removal (mech) by rubbing with a tissue. In both cases the fruit looked black to the eye, but mechanical removal produced a high-reflectance surface in most cases i.e. a specular reflectance measurement from the rubbed-smooth surface layer is measured here. Chloroform wax removal produced a matte surface, due to no smooth interface between the wax-impregnated waxy cuticle and the external extracuticular wax. b) Spectra from pigmented skin/juice of barberry fruits revealed by peeling the skin and measuring the pigmented underside of the fruit skin layer.

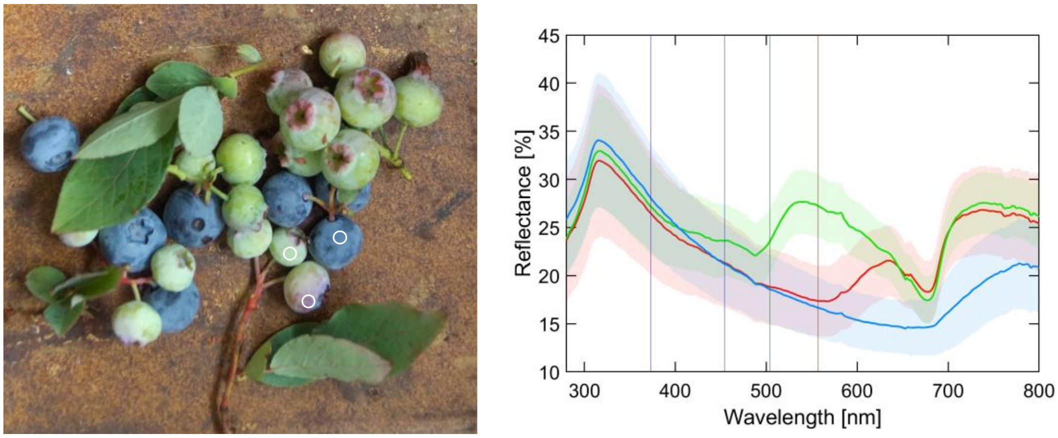

Fig. S3. Spectra from maturing blueberries. (A) Ripe and unripe blueberries coated in wax, white circles indicate characteristic colour patches appearing red, green and blue**. (B)** Spectra measured from immature (red and green) and mature (blue) fruits, showing the effect of bloom reflectance in each case, but in the unripe fruits, pigmentary colour is also visible, and in fact dominates the visual appearance. The solid lines indicate the mean reflectance of measurements from 37 fruits, the shaded areas show 1 standard deviation. The maximally sensitive absorption wavelength effective in the four blackbird colour vision cones are indicated by vertical lines, although it is important to note that the cones accept a wide range of wavelengths either side of their highest sensitivity.

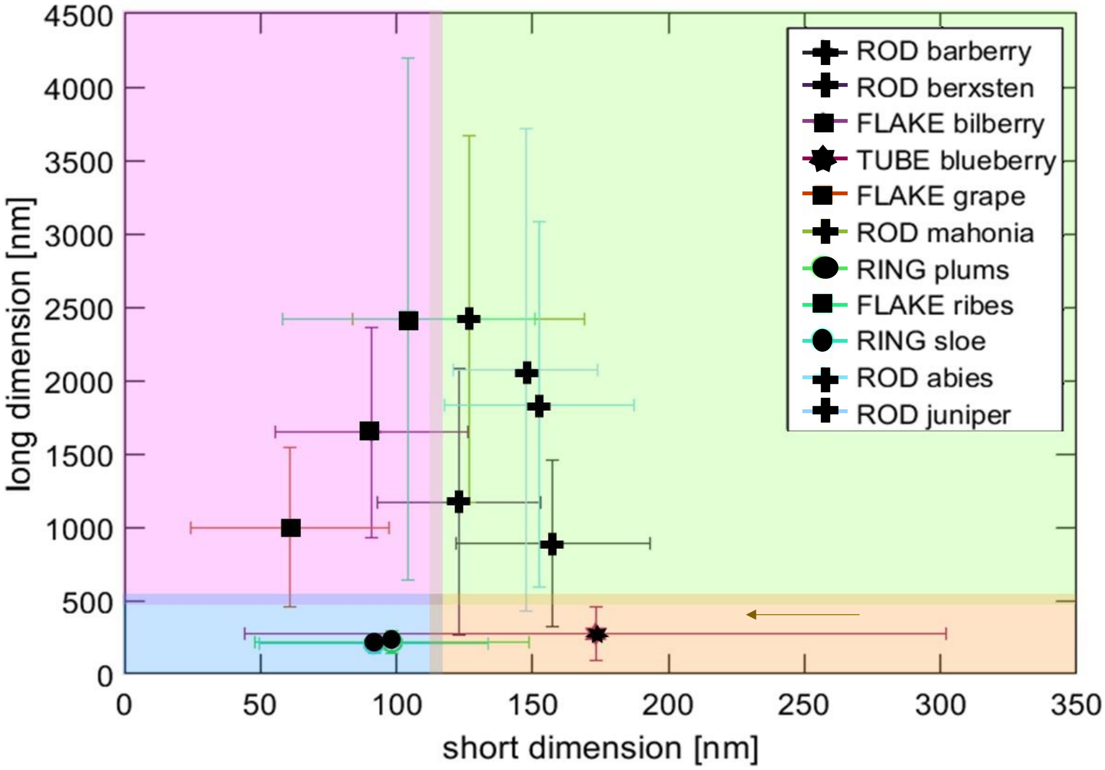

Fig. S4. Characteristic dimensions of wax particles. The photonic resonance length scale indicated is assuming close packing. 50 particles were measured from each fruit and the mean values ±95% confidence intervals plotted here. The colored quadrants correspond to the different types of shapes (flake, rod, ring, tube). The particle sizes are close to, or on ‘a photonic scale’ – at which coherent interference effects across appear. Assuming a refractive index of around 1.4 the optical length across a particle of dimension 120 nm would be 143 nm, or a double-length (ie. path difference for rays from front and back of the particle) of 285 nm.

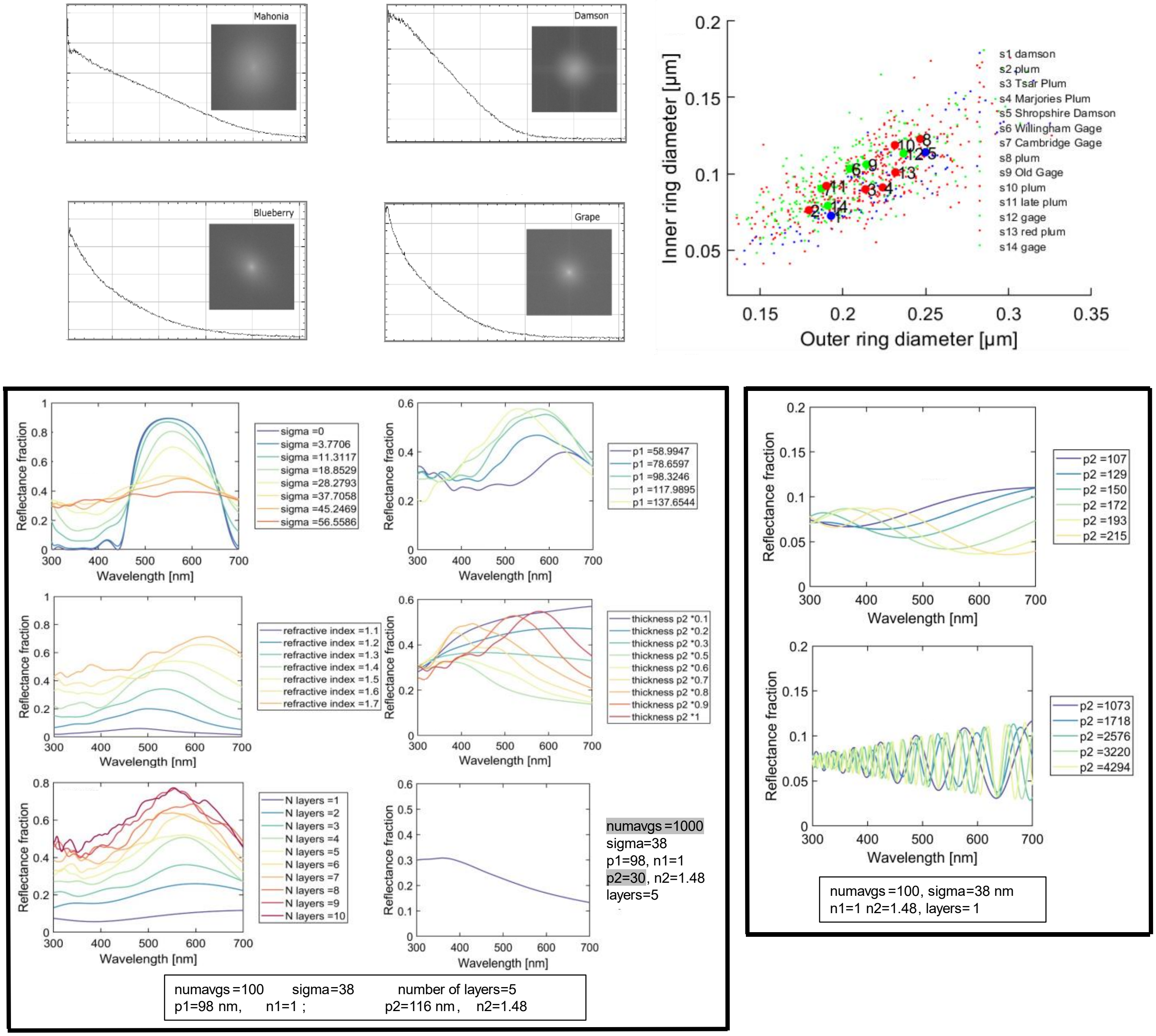

Fig. S5. Limitations on the development of 1D coherent interference model. (a) Spatial Fourier transforms of SEM images of wax particles. The plot shows a 1D integration around 360° from the centre of the 2D Fourier transform intensity plot, inset. **(i),** Mahonia (*Mahonia aquifolium*)**, (ii),** damson *(Prunus domestica subsp. Insititia)*, **(iii),** blueberry (*Vaccinium corymbosum*), **(iv),** grape *(Vitis vinifera)*. **(b)** Crystal dimensions are plotted for inner and outer ring diameters for a range of *Prunus* 'ring' particles.
(c) An averaged 1D transfer matrix model used to understand the effect of coherent reflectance from periodic boundaries, with a disorder parameter. The parameters used (layer thicknesses, corresponding to particle particle diameter, with contributions from wax and hole diameter, and standard deviation in this) are based off measurements of wax 'ring' particles as these are the most highly symmetric and packed particles observed across the bloom fruits. These parameters are then varied to show the spectral response in (**c.i-v).** Finally, a spectrum is specifically constructed to approximate the typical 'bloom spectrum profile'. This is the selection of 'closest fit' in which parameters are manipulated to produce a spectrum closest to the decreasing relationship between wavelength and intensity, whilst maintaining non-negligible ring thickness. Unfortunately, it should be noted however that this is not a good approximation to the measured ring dimensions. As can be observed in the parameter sweep plots, all attempts to construct a coherent interference with parameters closer to those measured produce instead a spectrum peaked in the visible range, which is not observed experimentally. The 1D multilayer was constructed by adapting the MATLAB script jreftran[2] to average over randomly distributed multilayers with known parameters of disorder. The

parameters given in the box underneath correspond to the ’default values’ in each model, except where the legend indicates parameters were changed in the sweep, thus: **(i)** change in standard deviation of layer thickness **(ii)** Change in refractive index of ‘wax layer’. **(iii)** Change in number of repeated layers **(iv)** Change in the thickness of the wax layer (ring wall thickness) **(v)** Change in air layer (hole diameter)**.**
(d.i & d.ii) Model a thin film, with thicknesses corresponding to both the thickness of an individual wax layer **(i)** and from a layer corresponding to the total wax coating, assuming no substructure. In both cases, it is clear that the thin film would have a broad band reflectance, or peak at longer wavelength. Neither show a prediction of higher reflectance at lower wavelengths.

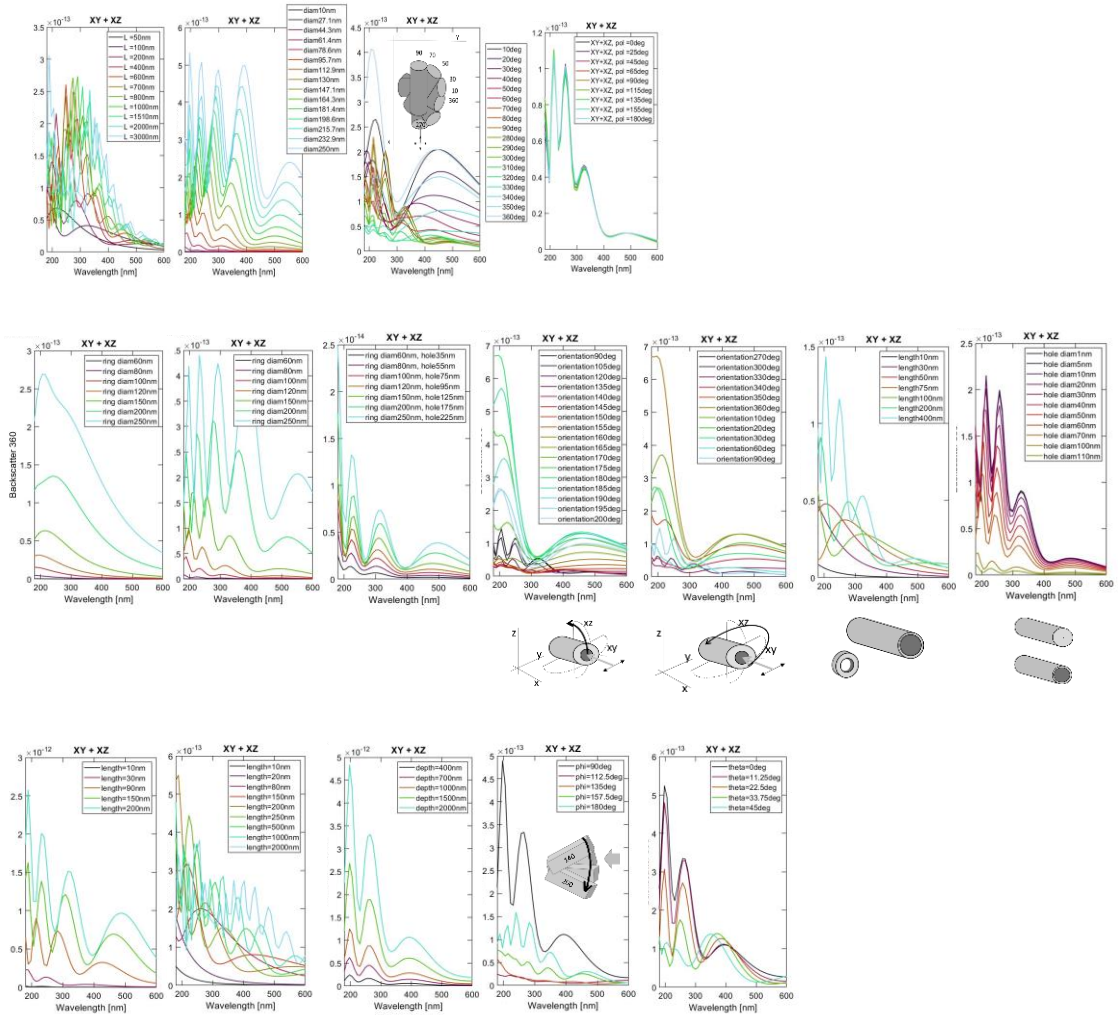

Fig. S6. Lumerical models varying parameters for rods (A), rings and tubes (B) and slabs (C). Unweighted averages were made over the sets of realistic parameters, assuming variation in particle shape and orientation according to that observed. The variables are listed in the legend text. The base dimensions and orientations of the particles are listed here. Rod: diameter 136.5 nm, length 400 nm, theta (angle from normal to optical axis) 280°. Ring: hole diameter 50 nm, diameter 136.5 nm, length 400 nm (Tube length 1100 nm), theta 280°. Slab: 600 nm x 300 nm x 50 nm, phi = 90° (angle of smallest face to optical axis), theta (angle of largest face from optical axis) = 10°.

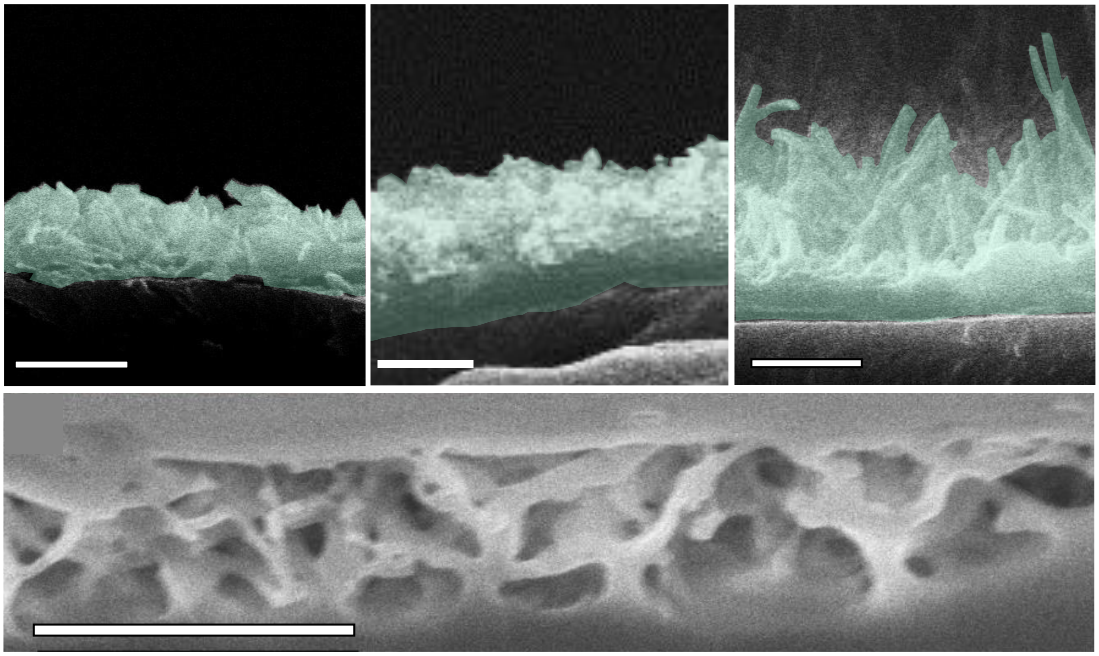

Fig. S7. SEM images of cross-sections through the scattering surfaces of unrelated species. (**A**) Grape. (**B**) Plum. **(C)** Mahonia showing layers of wax (indicated in blue) of 2 μm, 4.5 μm, 2.5 μm, 1.8 μm thickness. **(D)** shows a 45-degree angle view of a cross-section through blueberry wax cut by FIB, the top is coated with a layer of platinum. The scalebar is 2 μm in each image.

REFERENCES AND NOTES

1. Y. Yan, M. Dossett, S. D. Castellarin, Cuticular waxes affect fruit surface color in blueberries. *Plants People Planet* **5**, 736–751 (2023).

2. Kinoshita S., *Structural Colors in the Realm of Nature* (World Scientific Publishing Co., 2008), pp. 076401–076431.

3. I. Meusel, C. Neinhuis, C. Markstädter, W. Barthlott, Chemical composition and recrystallization of epicuticular waxes: Coiled rodlets and tubules. *Plant Biol.* **2**, 462–470 (2000).

4. A. Kelber, M. Vorobyev, Animal colour vision—Behavioural tests and physiological concepts. *Biol. Rev. Camb. Philos. Soc.* **78**, 81–118 (2003).

5. D. Osorio, M. Vorobyev, Colour vision as an adaptation to frugivory in primates. *Proc. Biol. Sci.* **263**, 593–599 (1996).

6. K. E. Stournaras, E. Lo, K. Böhning-Gaese, E. Cazetta, D. M. Dehling, M. Schleuning, M. C. Stoddard, M. J. Donoghue, R. O. Prum, H. Martin Schaefer, How colorful are fruits? Limited color diversity in fleshy fruits on local and global scales. *New Phytol.* **198**, 617–629 (2013).

7. P. R. Denish, J.-A. Fenger, R. Powers, G. T. Sigurdson, L. Grisanti, K. G. Guggenheim, S. Laporte, J. Li, T. Kondo, A. Magistrato, M. P. Moloney, M. Riley, M. Rusishvili, N. Ahmadiani, S. Baroni, O. Dangles, M. Giusti, T. M. Collins, J. Didzbalis, K. Yoshida, J. B. Siegel, R. J. Robbins, Discovery of a natural cyan blue: A unique food-sourced anthocyanin could replace synthetic brilliant blue. *Sci. Adv.* **7**, eabe7871 (2021).

8. R. M. Christie, Why is indigo blue? *Biotech. Histochem.* **82**, 51–56 (2007).

9. M. Shiono, N. Matsugaki, K. Takeda, Structure of the blue cornflower pigment. *Nature* **436**, 791 (2005).

10. S. J. Bloor, Deep blue anthocyanins from blue *Dianella* berries. *Phytochemistry* **58**, 923–927 (2001).

11. D. W. Lee, G. T. Taylor, A. K. Irvine, Structural fruit coloration in *Delarbrea michieana* (Araliaceae). *Int. J. Plant Sci.* **161**, 297–300 (2000).

12. D. W. Lee, Ultrastructural basis and function of iridescent blue colour of fruits in *Elaeocarpus*. *Nature* **349**, 260–262 (1991).

13. S. Vignolini, P. J. Rudall, A. V. Rowland, A. Reed, E. Moyroud, R. B. Faden, J. J. Baumberg, B. J. Glover, U. Steiner, Pointillist structural color in *Pollia* fruit. *Proc. Natl. Acad. Sci. U.S.A.* **109**, 15712–15715 (2012).

14. R. Middleton, E. Moyroud, P. J. Rudall, C. J. Prychid, M. Conejero, B. J. Glover, S. Vignolini, Using structural colour to track length scale of cell-wall layers in developing *Pollia japonica* fruits. *New Phytol.* **230**, 2327–2336 (2021).

15. S. Vignolini, T. Gregory, M. Kolle, A. Lethbridge, E. Moyroud, U. Steiner, B. J. Glover, P. Vukusic, P. J. Rudall, Structural colour from helicoidal cell-wall architecture in fruits of *Margaritaria nobilis*. *J. R. Soc. Interface* **13**, 20160645 (2016).

16. R. Middleton, M. Sinnott-Armstrong, Y. Ogawa, G. Jacucci, E. Moyroud, P. J. Rudall, C. Prychid, M. Conejero, B. J. Glover, M. J. Donoghue, S. Vignolini, *Viburnum tinus* fruits use lipids to produce metallic blue structural color. *Curr. Biol.* **30**, 3804–3810.e2 (2020).

17. M. A. Sinnott-Armstrong, R. Middleton, Y. Ogawa, G. Jacucci, E. Moyroud, B. J. Glover, P. J. Rudall, S. Vignolini, M. J. Donoghue, Multiple origins of lipid-based structural colors contribute to a gradient of fruit colors in *Viburnum* (Adoxaceae). *New Phytol.* **237**, 643–655 (2023).

18. M. A. Sinnott-Armstrong, Y. Ogawa, G. T. van de Kerkhof, S. Vignolini, S. D. Smith, Convergent evolution of disordered lipidic structural colour in the fruits of *Lantana strigocamara* (syn. *L. camara* hybrid cultivar). *New Phytol.* **235**, 898–906 (2022).

19. S. W. Chung, D. J. Yu, H. J. Lee, Changes in anthocyanidin and anthocyanin pigments in highbush blueberry (*Vaccinium corymbosum* cv. Bluecrop) fruits during ripening *Hortic. Environ. Biotechnol.* **57**, 424–430 (2016).

20. R. A. Moyer, K. E. Hummer, C. E. Finn, B. Frei, R. E. Wrolstad, Anthocyanins, phenolics, and antioxidant capacity in diverse small fruits: *Vaccinium*, *Rubus*, and *Ribes*. *J. Agric. Food Chem.* **50**, 519–525 (2002).

21. H. Bargel, K. Koch, Z. Cerman, C. Neinhuis, Structure-function relationships of the plant cuticle and cuticular waxes—A smart material? *Funct. Plant Biol.* **33**, 893–910 (2006).

22. W. Barthlott, C. Neinhuis, Purity of the sacred lotus, or escape from contamination in biological surfaces. *Planta* **202**, 1–8 (1997).

23. H. J. Ensikat, M. Boese, W. Mader, W. Barthlott, K. Koch, Crystallinity of plant epicuticular waxes: Electron and x-ray diffraction studies. *Chem. Phys. Lipids* **144**, 45–59 (2006).

24. B. Bhushan, Y. C. Jung, A. Niemietz, K. Koch, Lotus-like biomimetic hierarchical structures developed by the self-assembly of tubular plant waxes. *Langmuir* **25**, 1659–1666 (2009).

25. G. T. Van De Kerkhof, L. Schertel, R. N. Poon, G. Jacucci, B. J. Glover, S. Vignolini, Disordered wax platelets on *Tradescantia pallida* leaves create golden shine. *Faraday Discuss.* **223**, 207–215 (2020).

26. J. B. Clark, G. R. Lister, Photosynthetic action spectra of trees. *Plant Physiol.* **55**, 407–413 (1975).

27. R. H. Grant, G. M. Heisler, W. Gao, M. Jenks, Ultraviolet leaf reflectance of common urban trees and the prediction of reflectance from leaf surface characteristics. *Agric. For. Meteorol.* **120**, 127–139 (2003).

28. T. C. Vogelmann, Plant tissue optics. *Annu. Rev. Plant. Physiol. Plant. Mol. Biol.* **44**, 231–251 (1993).

29. M. Kerker, Blue skies and the Tyndall effect. *J. Chem. Educ.* **48**, 389 (1971).

30. J. R. Byers, Tyndall blue and surface white of tent caterpillars, *Malacosoma* SPP. *J. Insect Physiol.* **21**, 401–415 (1975).

31. J. Huxley, The coloration of *Papilio zalmoxis* and *P. antimachus*, and the discovery of Tyndall blue in butterflies. *Proc. R. Soc. B.* **193**, 441–453 (1976).

32. G. S. He, H.-Y. Qin, Q. Zheng, Rayleigh, Mie, and Tyndall scatterings of polystyrene microspheres in water: Wavelength, size, and angle dependences. *J. Appl. Phys.* **105**, 023110 (2009).

33. R. O. Prum, Blue integumentary structural colours in dragonflies (*Odonata*) are not produced by incoherent Tyndall scattering. *J. Exp. Biol.* **207**, 3999–4009 (2004).

34. K. Baek, Y. Kim, S. Mohd-Noor, J. K. Hyun, Mie resonant structural colors. *ACS Appl. Mater. Interfaces* **12**, 5300–5318 (2020).

35. T. Okazaki, H. Sugimoto, T. Hinamoto, M. Fujii, Color toning of mie resonant silicon nanoparticle color inks. *ACS Appl. Mater. Interfaces* **13**, 13613–13619 (2021).

36. V. Hwang, A. B. Stephenson, S. Barkley, S. Brandt, M. Xiao, J. Aizenberg, V. N. Manoharan, Designing angle-independent structural colors using Monte Carlo simulations of multiple scattering. *Proc. Natl. Acad. Sci. U.S.A.* **118**, e2015551118 (2021).

37. G. Jacucci, S. Vignolini, L. Schertel, The limitations of extending nature's color palette in correlated, disordered systems. *Proc. Natl. Acad. Sci. U.S.A.* **117**, 23345–23349 (2020).

38. V. Hwang, A. B. Stephenson, S. Magkiriadou, J.-G. Park, V. N. Manoharan, Effects of multiple scattering on angle-independent structural color in disordered colloidal materials. *Phys. Rev. E* **101**, 012614 (2020).

39. R. M. Parker, T. H. Zhao, B. Frka-Petesic, S. Vignolini, Cellulose photonic pigments. *Nat. Commun.* **13**, 3378 (2022).

40. K. Koch, W. Barthlott, Plant epicuticular waxes: Chemistry, form, self-assembly and function. *Nat. Prod. Commun.* **1**, 1067–1072 (2006).

41. K. Koch, A. Dommisse, A. Niemietz, W. Barthlott, K. Wandelt. Nanostructure of epicuticular plant waxes: Self-assembly of wax tubules. *Surf. Sci.* **603**, 1961–1968 (2009).

42. W. Barthlott, C. Neinhuis, D. Cutler, F. Ditsch, I. Meusel, I. Theisen, H. Wilhelmi, Classification and terminology of plant epicuticular waxes. *Bot. J. Linn. Soc.* **126**, 237–260 (1998).

43. H. Noh, S. F. Liew, V. Saranathan, S. G. J. J. Mochrie, R. O. Prum, E. R. Dufresne, H. Cao, How noniridescent colors are generated by quasi-ordered structures of bird feathers. *Adv. Mater.* **22**, 2871–2880 (2010).

44. G. Shang, M. Eich, A. Petrov, Photonic glass based structural color. *APL Photonics* **5**, 060901.

45. E. Marx, G. W. Mulholland, Size and refractive index determination of single polystyrene spheres. *J. Res. Natl. Bur. Stand.* **88**, 321–338 (1983).

46. R. Jetter, S. Schäffer, M. Riederer. Leaf cuticular waxes are arranged in chemically and mechanically distinct layers: Evidence from *Prunus laurocerasus* L. *Plant Cell Environ.* **23**, 619–628 (2000).

47. T. W. Mulroy. Spectral properties of heavily glaucous and non-glaucous leaves of a succulent rosette-plant. *Oecologia* **38**, 349–357 (1979).

48. E. Moyroud, T. Wenzel, R. Middleton, P. J. Rudall, H. Banks, A. Reed, G. Mellers, P. Killoran, M M. Westwood, U. Steiner, S. Vignolini, B. J. Glover, Disorder in convergent floral nanostructures enhances signalling to bees. *Nature* **550**, 469–474 (2017).

49. E. Cazetta, H. M. Schaefer, M. Galetti, Why are fruits colorful? the relative importance of achromatic and chromatic contrasts for detection by birds. *Evol. Ecol.* **23**, 233–244 (2009).

50. M. F. Willson, C. J. Whelan, Ultraviolet reflectance of fruits of vertebrate-dispersed plants. *Oikos* **55**, 341 (1989).

51. H. Siitari, J. Honkavaara, J. Viitala, Ultraviolet reflection of berries attracts foraging birds. A laboratory study with redwings (*Turdus iliacus*) and bilberries (*Vaccinium myrtillus*). *Proc. Soc. B. Biol. Sci.* **266**, 2125–2129 (1999).

52. Y. Han, Z. Meng, Y. Wu, S. Zhang, S. Wu, Structural colored fabrics with brilliant colors, low angle dependence, and high color fastness based on the Mie scattering of Cu2O spheres. *ACS Appl. Mater. Interfaces* **13**, 57796–57802 (2021).

53. L. Maiwald, S. Lang, D. Jalas, H. Renner, A. Y. Petrov, M. Eich, Ewald sphere construction for structural colors. *Opt. Express* **26**, 11352–11365 (2018).

54. B. D. Wilts, X. Sheng, M. Holler, A. Diaz, M. Guizar-Sicairos, J. Raabe, R. Hoppe, S.-H. Liu, R. Langford, O. D. Onelli, D. Chen, S. Torquato, U. Steiner, C. G. Schroer, S. Vignolini, A. Sepe, Evolutionary-optimized photonic network structure in white beetle wing scales. *Adv. Mater.* **30**, 1702057 (2018).

55. J. D. Forster, H. Noh, S. F. Liew, V. Saranathan, C. F. Schreck, L. Yang, J.-G. Park, R. O. Prum, S. G. J. Mochrie, C. S. O'Hern, H. Cao, E. R. Dufresne, Biomimetic isotropic nanostructures for structural coloration. *Adv. Mater.* **22**, 2939–2944 (2010).

56. E. S. A. Goerlitzer, R. N. Klupp Taylor, N. Vogel, Bioinspired photonic pigments from colloidal self-assembly. *Adv. Mater.* **30**, e1706654 (2018).

57. K. Koch, W. Barthlott, S. Koch, A. Hommes, K. Wandelt, W. Mamdouh, S De-Feyter, P. Broekmann, Structural analysis of wheat wax (*Triticum aestivum*, c.v. "Naturastar" L.): From the molecular level to three dimensional crystals. *Planta* **223**, 258–270 (2006).

58. R. Maia, H. Gruson, J. A. Endler, T. E. White, pavo2: New tools for the spectral and spatial analysis of colour inr. *Methods Ecol. Evol.* **10**, 1097–1107 (2019).

59. N. S. Hart, J. C. Partridge, I. C. Cuthill, Visual pigments, oil droplets and cone photoreceptor distribution in the European starling (*Sturnus vulgaris*). *J. Exp. Biol.* **201**, 1433–1446 (1998).

60. N. S. Hart, M. Vorobyev, Modelling oil droplet absorption spectra and spectral sensitivities of bird cone photoreceptors. *J. Comp. Physiol. A Neuroethol. Sens. Neural Behav. Physiol.* **191**, 381–392 (2005).

61. S. Kumar, A. P. Bhondekar, P. Jain, S. Bagchi, A. Sharma, R. Kumar, S. Mishra, Artificial lipid membrane: Surface modification and effect in taste sensing. *IOP Conf. Ser. Mater. Sci. Eng.* **360**, 012039 (2018).

62. M. Vorobyev, D. Osorio, Receptor noise as a determinant of colour thresholds. *Proc. Soc. B Biol. Sci.* **265**, 351–358 (1998).

63. W. Chu, H. Gao, S. Cao, X. Fang, H. Chen, S. Xiao. Composition and morphology of cuticular wax in blueberry (Vaccinium spp.) fruits. *Food Chem.* **219**, 436–442 (2017).
